# An *rpoB* Sequence Type Network as a Framework for the Evolutionary Investigation of *Clostridium perfringens*

**DOI:** 10.3390/microorganisms13122768

**Published:** 2025-12-04

**Authors:** Sun-Min Ahn, Seungeun Son, Yongwoo Son, So-Jeong Lim, Danil Kim, Kang-Seuk Choi, Hyuk-Joon Kwon

**Affiliations:** 1 Laboratory of Poultry Medicine, Department of Farm Animal Medicine, College of Veterinary Medicine and BK21 PLUS for Veterinary Science, Seoul National University, Seoul 88026, Republic of Korea; vicky.ahn@snu.ac.kr; 2 Research Institute for Veterinary Science, College of Veterinary Medicine, Seoul National University, Seoul 08826, Republic of Korea; arbre04@snu.ac.kr (S.S.); sojing@snu.ac.kr (S.-J.L.); danilkim@snu.ac.kr (D.K.); 3 Laboratory of Avian Diseases, College of Veterinary Medicine, Seoul National University, Seoul 08826, Republic of Korea; 4 Wildlife Health Laboratory, College of Veterinary Medicine, Konkuk University, Seoul 05029, Republic of Korea; yongwooson@konkuk.ac.kr; 5 Farm Animal Clinical Training and Research Center (FACTRC), GBST (Green Bio Science and Technology), Seoul National University, Seoul 08826, Republic of Korea; 6 Department of Farm Animal Medicine, Seoul National University, Pyeongchang-gun 25354, Republic of Korea; 7 GeNiner Inc., Seoul 08826, Republic of Korea

**Keywords:** *Clostridium perfringens*, *rpoB*, network analysis, comparative genomics, evolution, interspecies transmission

## Abstract

*Clostridium perfringens* is an opportunistic Gram-positive bacterium that causes necrotic enteritis and other severe infections in animals, as well as food poisoning in humans. In this study, we introduce a framework consisting of *rpoB* sequence typing (RSTing) and network analysis to investigate the evolutionary trajectories of *C. perfringens*. By analyzing 319 *rpoB* sequences—300 from public databases and 19 newly sequenced isolates from chicken and cattle sources—we identified 84 *rpoB* sequence types (RSTs). Among them, the early emerging RST 1-1 was the most prevalent (21.3%), while the putative ancestral type, RST 0, was the fifth most common (4.7%). The high RST diversity and the predominance of RST 1-1, mainly from chickens, suggest that chickens may serve as an important reservoir. By integrating virulence gene profiling, MLST, and comparative genomics, we separated identical RSTs into distinct genotypes and uncovered genomic evidence of possible interspecies transmission between chickens and cattle, two major food-producing species. These findings indicate that RSTing provides a useful complementary approach to investigating the evolutionary and epidemiological dynamics of *C. perfringens*.

## 1. Introduction

*Clostridium perfringens* (*C. perfringens*) is a Gram-positive, spore-forming bacterium within the Firmicutes phylum [1]. It is a zoonotic pathogen that is capable of causing various diseases depending on the toxins that it harbors [2]. Major toxin types in *C. perfringens* include alpha, beta, epsilon, iota, enterotoxin (CPE), and necrotic enteritis B-like toxin (NetB), which are responsible for various diseases in animals and humans [2,3]. These toxins enable *C. perfringens* to act as an opportunistic pathogen, being widely persistent as an environmental contaminant [3,4]. In humans, it is a major cause of food poisoning, while, in animals, it is associated with necrotic enteritis (NE), hemorrhagic bowel syndrome, and gas gangrene [5,6,7]. Notably, *C. perfringens* represents a significant economic burden for the broiler industry, especially following the ban on antimicrobial growth promoters (AGPs), which were previously used to enhance growth and feed efficiency [8].

Given their wide host range, clinical impact, and genetic variability, the accurate classification and evolutionary tracking of *C. perfringens* strains are essential for epidemiological surveillance and disease control. Traditional methods such as multi-locus sequence typing (MLST), 16S rRNA sequencing, and PFGE each have limitations in terms of cost, resolution, or evolutionary insight [9]. Recent advances in pan-genome and whole-genome approaches have provided deeper insights into genetic diversity and mobile element dynamics in *C. perfringens* [10,11]. However, simpler and more universally applicable genetic markers remain highly desirable.

RNA polymerase, which is essential for the synthesis of vital RNA, mRNA, rRNA, and tRNA in all living microorganisms, serves as a critical link among the RNA, DNA, and protein domains [12,13]. The RNA polymerase beta subunit gene (*rpoB*) has been successfully used as a phylogenetic marker; in conjunction with network analysis, it has been applied to understand the evolutionary trajectory of *E. coli* [13,14,15]. The progenitor–progeny relationships, which were determined through *rpoB* sequence typing (RSTing) and network analysis, were verified by the high genomic coverage and identity obtained through comparative genomics [13,16]. The universal presence of *rpoB* in bacteria and its low likelihood of undergoing horizontal gene transfer or recombination make it an ideal candidate with which to trace evolutionary changes. In addition, molecular prophage typing (mPPTing) and CRISPR profiling have provided insights into the interactions between temperate phages and foreign plasmids, which facilitate the transfer of genes related to pathogenicity [13].

In this study, we established a new RSTing scheme and performed a network analysis of *rpoB* sequence types to infer the evolutionary trajectory of *C. perfringens*. The presence of the progenitor (RST 0) and the high prevalence of RST 1-1 suggest a relatively slow rate of evolution in this species. Additionally, the distribution of various RSTs among avian and ruminant hosts suggests that these animals may serve as major natural reservoirs. Such reservoirs may, in turn, pose a significant risk regarding transmission to humans. Comparative genomic analysis further validated the evolutionary relationships inferred from the RSTing network. When combined with outcomes derived from mPPTing, CRISPR profiling, and conventional MLST data, these findings provide deeper evolutionary and molecular-epidemiological insights into the evolutionary traits and interspecies transmission of *C. perfringens*.

## 2. Materials and Methods

### 2.1. Bacterial Isolation and Identification

In this study, we analyzed nine isolates from chickens and ten isolates from cattle. Chicken isolates were collected from broiler, layer, and breeder chickens across different farms in Republic of Korea during 2020 and 2021, while ruminant-derived isolates were collected from different animals at different time points within the same farm. Carcasses from the sampled chickens were necropsied at the School of Veterinary Medicine, Avian Disease Laboratory, Seoul National University (Seoul, Republic of Korea). Based on the necropsy findings, samples (approximately 1 cm × 1 cm of cecal tissue and feces) were collected and processed for pathogen detection (Supplementary Table S1) [17,18,19,20,21,22]. *C. perfringens* isolates from ruminants were obtained from Holstein cows provided by the College of Agricultural and Life Sciences Animal Farm at Seoul National University (Pyeongchang, Republic of Korea). All samples were cultured anaerobically on tryptose sulfite cycloserine agar (Duchefa, Haarlem, The Netherlands) and cooked meat medium using GasPak™ EZ pouch systems (BD Biosciences, Franklin Lakes, NJ, USA). Colonies isolated from chickens were initially identified using the MALDI-TOF Biotyper system (Bruker Daltonics, Bremen, Germany), while samples from cattle were preliminarily identified using the VITEK^®^ 2 system (bioMérieux, Marcy-l’Étoile, France).

### 2.2. Primers, PCR, and Sequencing

Genomic DNA was extracted using the G-spin™ Genomic DNA Extraction Kit (Intron Biotechnology, Seongnam, Republic of Korea). Toxin genes, the housekeeping genes used for MLST, *rpoB* and avian pathogen-associated target genes were amplified through PCR using both previously published and newly designed primers (Supplementary Table S1) [7,23,24,25,26,27,28]. For *rpoB* consensus construction, a total of 300 sequences, including both whole and fragmented sequences, were retrieved from the GenBank NCBI database and PubMLST for subsequent analysis (accessed on 12 May 2021) (Supplementary Table S3). Regarding the NCBI database, the search query “*Clostridium perfringens rpoB*” was used within the nucleotide database, retrieving entries labeled as “complete genome”, “contig”, or “whole-genome shotgun sequence” that contained the *rpoB* locus. In PubMLST, an organism search for *C. perfringens* was performed, and genome entries containing *rpoB* were extracted. Redundant entries and truncated *rpoB* sequences that failed to span the complete coding region were excluded from the analysis. Nineteen new sequences obtained in this study were included in the analysis, bringing the total to 319 sequences. Sequence alignment was performed using MUSCLE within the UGENE software (version 40) [29].

### 2.3. Multi-Locus Sequence Typing, rpoB Sequence Typing, Network Analysis, and Phylogenetic Analysis

Multi-locus sequence typing (MLST) was performed on the 19 newly sequenced strains, as previously described [26]. In parallel, a total of 319 *rpoB* sequences—comprising 300 from public databases and the 19 newly sequenced strains—were converted into haplotypes based on SNPs using DnaSP (version 6.0) [30]. RST numbers were assigned according to nucleotide differences relative to the consensus, following established protocols [16,31,32]. The resulting haplotypes were then used for network construction. Median joining networks were generated using PopArt (version 1.7) [33], and haplotypes were subsequently labeled using Adobe Illustrator (version 27.0, Adobe Inc., San Jose, CA, USA).

After alignment using MUSCLE within UGENE, full-length *rpoB* nucleotide sequences were analyzed for potential recombination events using RDP (v4.101) under the default parameters. No recombination events were detected, indicating that the dataset was suitable for phylogenetic inference. A model-based maximum likelihood (ML) tree was subsequently constructed using IQ-TREE (v3.0.1), with the optimal substitution model selected using ModelFinder Plus (integrated into IQ-TREE). Branch supports were calculated from 1000 SH-aLRT and 1000 ultrafast bootstrap replicates, and nodes with both SH-aLRT ≥ 80% and UFBoot ≥ 95% were regarded as well supported. The final tree was visualized in iTOL (Interactive Tree of Life, version 6) [34]. The resulting tree was exported in Newick format, with RST 0 designated as the outgroup.

### 2.4. Whole-Genome Sequencing and Comparative Genomics

Whole-genome sequencing (WGS) was performed on five selected strains: SNUVG (cattle, RST 0), SNU11038 (cattle, RST 1-1), SNU20021 (chicken, RST 1-1), SNU61035 (cattle, RST 2-17), and SNU21005 (chicken, RST 2-17). This was achieved using a hybrid sequencing approach combining a PacBio long-read platform (SMRTbell library) and an Illumina short-read platform (100 bp paired end, TruSeq library). De novo assembly was performed using PacBio long reads as the primary dataset, with Illumina reads for error correction. Assembly quality was validated by K-mer analysis, assessing the read depth, GC content distribution, and sequence accuracy. Chromosomal contigs were identified, and plasmid sequences with low query coverage were excluded. Circular plasmid contigs were identified when the 5′ and 3′ ends were connected. Genome annotation was conducted using the DFAST pipeline (version 1.2.0, accessed on 4 October 2022) [35], which enables automated gene prediction and homology-based annotation against public databases to identify coding sequences (CDSs), transfer RNA (tRNA) genes, and ribosomal RNA (rRNA) genes for each contig.

Genomic islands were predicted using IslandViewer 4 [36], and plasmid insertion sites were analyzed via ISFinder (version 2.0, accessed on 1 December 2022) [37]. OriT DB (version 2.0) was used to analyze plasmids that harbored the *cpb2* gene. For the whole-genome comparison of RST 1-1, non-fragmented complete sequences available in the NCBI GenBank database were selected for analysis.

### 2.5. Prophage and CRISPR Analysis

For in silico mPPT, large terminase coding regions of reference strains were analyzed and classified into groups based on sequence similarity, bringing the total to 24 binary variables (Supplementary Tables S1 and S6). Prophage typing was subsequently performed via a BLAST search (version 2.13.0, accessed on 22 August 2022) of the nucleotide sequences of gene fragments from complete genome sequences, as previously described [13]. CRISPR arrays were identified using the CRISPR Recognition Tool (version 1.1) [38] and annotated using CRISPRTarget and BLASTN (version 2.13.0, accessed on 22 August 2022). Spacer–target matches were detected without applying strict identity or coverage thresholds so as to comprehensively capture both exact and near-exact homologs; sequence identities ranged from 77.8% to 100% (Supplementary Table S7). CRISPR-Cas system types were determined for 47 GenBank sequences using CRISPRCasTyper (version 1.8.0) [39].

## 3. Results

### 3.1. Toxinotypes of Clostridium Perfringens Strains

The conventional toxinotyping scheme, based on the profiles of major toxin genes—*cpa/plc* (alpha toxin), *cpb* (beta toxin), *etx* (epsilon toxin), and *iap* (iota toxin)—enables the classification of *C. perfringens* into types A to E. Recently, an expanded classification scheme has been proposed, incorporating the *cpe* (enterotoxin) and *netB* genes and extending the toxinotypes to include types F and G [2]. In this study, we characterized nine chicken-derived and ten cattle-derived *C. perfringens* strains. All chicken strains and eight of the cattle strains harbored only the *cpa/plc* gene and were classified as toxinotype A. Two cattle strains, SNU513 and SNU7253-1, carried both *cpa/plc* and *iap* and were therefore classified as toxinotype E (Table 1). None of the isolates harbored the enterotoxin genes *cpe*, *becA*, or *becB*, which are associated with human gastroenteritis [25,40]. The *netB* and *tpeL* toxin genes, which are critical for the pathogenesis of necrotic enteritis in poultry, were also absent. Similarly, sialidase genes (*nanH*, *nanI*, and *nanJ*) and hyaluronidase genes (*nagH*, *nagI*, *nagJ*, *nagK*, and *nagL*) were not detected in any isolates. In contrast, all isolates were positive for the *pfoA* (θ toxin) and *colA* (κ toxin) genes, while the β2 toxin gene (*cpb2*) was absent only in strains SNU21005 and SNU61035 (Supplementary Table S2).

### 3.2. Co-Infections Involving C. perfringens and Other Avian Pathogens

In addition to *C. perfringens*, co-infections with other avian pathogens were detected in all nine necropsy cases (Table 1). Among the eight pathogenic viruses screened, fowl adenovirus (FAdV, 7/9), infectious bronchitis virus (IBV, 7/9), infectious bursal disease virus (IBDV, 4/9), and chicken anemia virus (CAV, 3/9) were the most frequently identified. Avian pathogenic *Escherichia coli* (APEC) was also a common bacterial pathogen, detected in four out of nine cases. Two or more immunosuppressive viruses, including CAV, FAdV, IBDV, and avian leukosis virus (ALV), were identified in every case. These findings indicate that *C. perfringens* was not the sole etiologic agent and suggest that co-infections with immunosuppressive viruses may have increased the disease severity and contributed to the pathological outcomes observed.

### 3.3. RSTing, MLST, and Evolutionary Trajectory of C. perfringens

A total of 300 *rpoB* sequences of *C. perfringens* from diverse host sources were obtained from public databases, including whole-genome sequences, partial sequences, and contigs. In addition, we determined the complete *rpoB* coding sequences (3705 nucleotides) for 19 avian- and cattle-derived *C. perfringens* strains (GenBank accession numbers LC885342–LC885355). Among these, 14 sequences were obtained via targeted sequencing in this study, while the remaining five were extracted from whole-genome sequences generated as part of this work. In total, 319 *rpoB* sequences (300 from public databases and 19 newly sequenced in this study) were used to generate a consensus sequence (Supplementary Table S3). RST numbers were assigned based on nucleotide differences relative to this consensus sequence, designated as RST 0. The second number in each RST denotes the specific position of variation, following previously established methods [9,13].

Overall, the 319 sequences were classified into 84 *rpoB* sequence types (RSTs), ranging from RST 0 to RST 17-1. The most prevalent type was RST 1-1, accounting for 21.3% of all sequences, followed by RST 11-1 (8.2%), RST 2-4 (5.6%), RST 8-3 (5.0%), and RST 0 (4.7%) (Supplementary Tables S3 and S4). Among the 319 sequences, the most common type of host origin was avian (110 sequences, 34.5%), followed by human (49 sequences, 15.4%), ruminant (48 sequences, 15.0%), environmental (42 sequences, 13.2%), unknown (22 sequences, 6.9%), canine (17 sequences, 5.3%), equine (16 sequences, 5.0%), porcine (6 sequences, 1.9%), camelid (5 sequences, 1.6%), murine (2 sequences, 0.6%), leporine (1 sequence, 0.3%), and mustelid (1 sequence, 0.3%). Notably, the predominant RST 1-1 was primarily of avian origin (34 of 68 sequences, 50.0%), followed by environmental (14.7%), human (11.8%), ruminant (8.8%), equine (7.4%), unknown (5.9%), and canine (1.5%) sources (Supplementary Table S4). Furthermore, avian-derived isolates exhibited the highest RST diversity, comprising 30 types (35.7%), followed by ruminant-derived (27 types, 32.1%) and human-derived (22 types, 26.2%) isolates. Among the chicken-derived isolates in our study, five were classified as RST 1-1, two as RST 2-17, one as RST 4-1, and one as RST 2-16. The ten cattle-derived isolates were classified as RST 0 (two isolates), RST 1-1 (three isolates), RST 1-4 (one isolate), RST 2-17 (one isolate), RST 5-9 (two isolates), and RST 7-2 (one isolate) (Table 1).

To compare RSTing and MLST in terms of correlation and resolution, we performed MLST on chicken- and cattle-derived isolates (Table 1 and Table S5). Among the 19 isolates analyzed, 9 were assigned to distinct STs: ST885 (SNUVG; RST 0), ST587 (SNU21008; RST 2-16), ST179 (SNU21004; RST 4-1), ST323 (SNU20021; RST 1-1), ST21 (SNU20055; RST 1-1), ST294 (SNU11038; RST 1-1), ST277 (SNU21005 and SNU61035; both RST 2-17), and ST601 (SNU124; RST 7-2). Four isolates could not be assigned to known STs and were therefore designated as UT1 (SNU21002; RST 1-1), UT2 (SNU17037; RST 1-1), UT3 (SNU21011; RST 1-1), and UT4 (SNU21012; RST 2-17). For five isolates, STs could not be determined because amplicons were missing for one or two of the eight housekeeping genes; these were designated as iUT1 (SNU1045; RST 0), iUT2 (SNU21006; RST 1-1), iUT3 (SNU11040; RST 1-4), and iUT4 (SNU7253-1 and SNU513; both RST 5-9). In addition, one isolate remained undetermined due to the absence of PCR bands. This failure was most likely due to primer mismatches in certain housekeeping gene regions, rather than poor DNA quality, highlighting a practical limitation of the current MLST primers regarding diverse *C. perfringens* lineages. Interestingly, MLST enabled the differentiation of all RST 1-1 isolates from one another, and SNU21012 was clearly distinguished from the other RST 2-17 isolates (SNU21005 and SNU61035). Notably, an analysis of only three housekeeping genes—*groEL*, *sod*, and *sigK*—was sufficient to distinguish all RST 1-1 and RST 2-17 isolates, suggesting that a simplified MLST scheme may be feasible following RSTing.

**Figure 1:**
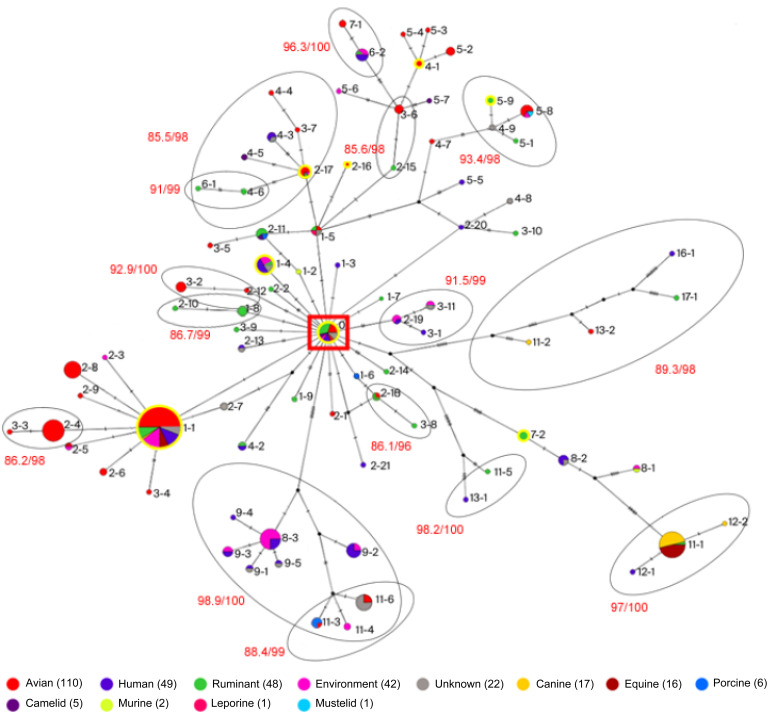
Spanning tree obtained from *rpoB* haplotype analysis. Sequences obtained in this study are outlined with yellow borders. Interspecies linkages are indicated, and stepwise RST spanning events (e.g., RST X–Y, where X represents the major type and Y the position of the variant) are labeled. The putative ancestral type, RST 0, is marked with a red square. Circular clustering in the network corresponds to well-supported phylogenetic relationships (SH-aLRT ≥ 80% and UFBoot ≥ 95%). The same high-support nodes are labeled in red on the phylogenetic tree (Supplementary Figure S1) and enclosed with gray circles in the network. Where multiple RSTs exhibit sequential similarity, circles are shown only between the most representative pair (e.g., RSTs 2-15 and 3-6) for visual clarity.

To investigate the evolutionary trajectories and progenitor–progeny relationships among RSTs, a median joining network analysis was performed (Figure 1). The network revealed 21 distinct branches originating from the common ancestral type, RST 0, each representing a different evolutionary pathway. Most terminal RSTs were classified as RST 5 or below, indicating relatively limited divergence from the progenitor. However, some lineages extended further to types such as RST 11, RST 12, and RST 17. The network illustrated direct progenitor–progeny relationships between RSTs. Based on the accumulation patterns of single-nucleotide polymorphisms (SNPs) in the *rpoB* gene, RSTs 2-3, 2-4/3-3, 2-5, 2-6, 2-8, 2-9, and 3-4 were inferred to be descendants of RST 1-1. Similarly, RSTs 9-1, 9-3, 9-4, and 9-5 were descended from RST 8-3, while RSTs 12-1 and 12-2 originated from RST 11-1. In addition, RST 2-17, which evolved from RST 1-5, was inferred to be the progenitor of RSTs 3-7/4-4, 4-3, 4-5, and 4-6/6-1. This evolutionary inference was further supported by the phylogenetic analysis. A maximum likelihood phylogenetic tree based on representative *rpoB* sequences of each RST revealed clustering patterns that were largely consistent with the RST network. Major branches were strongly supported (SH-aLRT ≥ 80%, UFBoot ≥ 95%), and similar clade structures were reflected in the network topology (Figure 1 and Figure S1). This concordance suggests that RSTing can complement conventional phylogenetic approaches in elucidating the evolutionary diversification of *C. perfringens*.

### 3.4. General Genomic Information for Five Representative C. perfringens Strains from RST 0, RST 1-1, and RST 2-17

Although numerous shotgun genome sequences of *Clostridium perfringens* are publicly available, complete genome sequences are still limited, and they are insufficient to support comprehensive comparative genomic analyses. To facilitate such analyses, and to characterize the key RST groups identified in this study, we selected five representative strains for whole-genome sequencing: SNUVG (cattle RST 0); SNU20021 and SNU11038 (chicken and cattle RST 1-1, respectively); and SNU21005 and SNU61035 (chicken and cattle RST 2-17, respectively) (Table 2). These genomes were sequenced de novo and annotated using the DFAST pipeline, followed by a comparison with the reference strain, ATCC 13124 [41].

Table 2 summarizes basic genomic features, including the genome size, GC content, number of coding sequences (CDSs), rRNAs, tRNAs, CRISPR arrays, coding density (%), plasmids, and genomic islands. Among the five complete genomes, the size ranged from 3,222,972 bp (SNU11038, RST 1-1) to 3,635,260 bp (SNU21005, RST 2-17), with GC content between 28.3% and 28.5%. The number of predicted CDSs varied from 2835 in SNU11038 to 3362 in SNU21005. Each strain was also assigned a sequence type (ST) according to the MLST results. Furthermore, structural genome elements such as mPPT (none or mPPT 15), CRISPR arrays (0 to 2), plasmids (1 to 5), and genomic islands (2 to 9) were identified, providing insights into the genetic variability across these representative strains.

### 3.5. Assessment of Evolutionary Relationships Between RSTs Based on Genome Coverage and Nucleotide Identity

**Figure 2:**
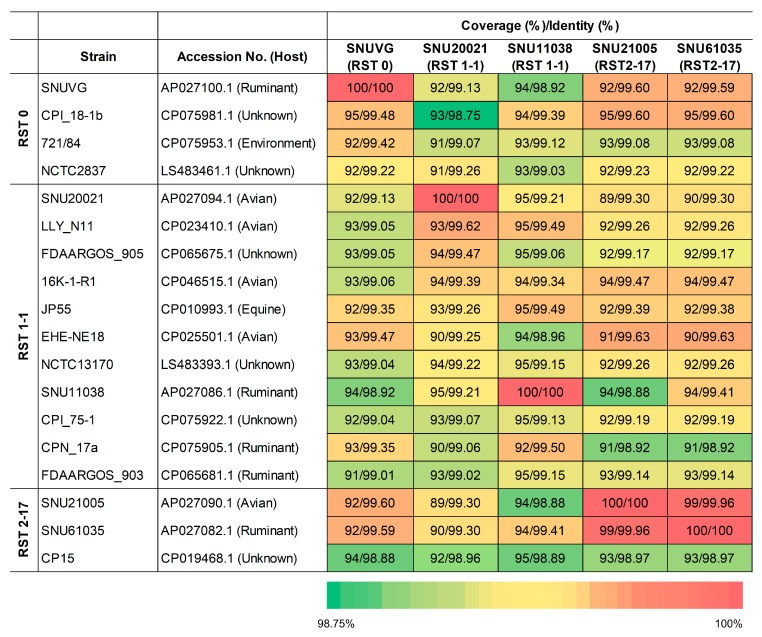
Percent coverage and identity (%) between RST 0, RST 1-1, and RST 2-17 strains.

The whole-genome sequences of *C. perfringens* strains were compared to assess their genome coverage and nucleotide identity (Figure 2). Within the public database, the SNUVG strain (RST 0) showed the highest similarity (95% coverage/99.48% identity) to another RST 0 strain, CPI_18-1b, and also displayed comparably high similarity to an RST 1-1 strain, EHE-NE18 (93.0%/99.47%). Our RST 2-17 strains, SNU21005 and SNU61035, also exhibited high nucleotide identity (99.60 and 99.59%, respectively) but comparatively lower genome coverage (92%).

Among the RST 1-1 strains, SNU20021 and SNU11038 shared 95% coverage/99.21% identity; however, even higher nucleotide identity was observed with other RST 1-1 strains, including LLY_N11 (99.62%) for SNU20021 and CPN_17a (99.50%) for SNU11038. The two RST 2-17 strains, SNU21005 and SNU61035, were nearly identical, with 99% coverage and 99.96% nucleotide identity, suggesting a close genetic relationship and potential interspecies transmission. In contrast, these two strains showed lower similarity to another RST 2-17 strain, CP15 (93%/98.97%), but unexpectedly higher similarity to the RST 0 strain CPI_18-1b (95%/99.60%).

**Figure 3:**
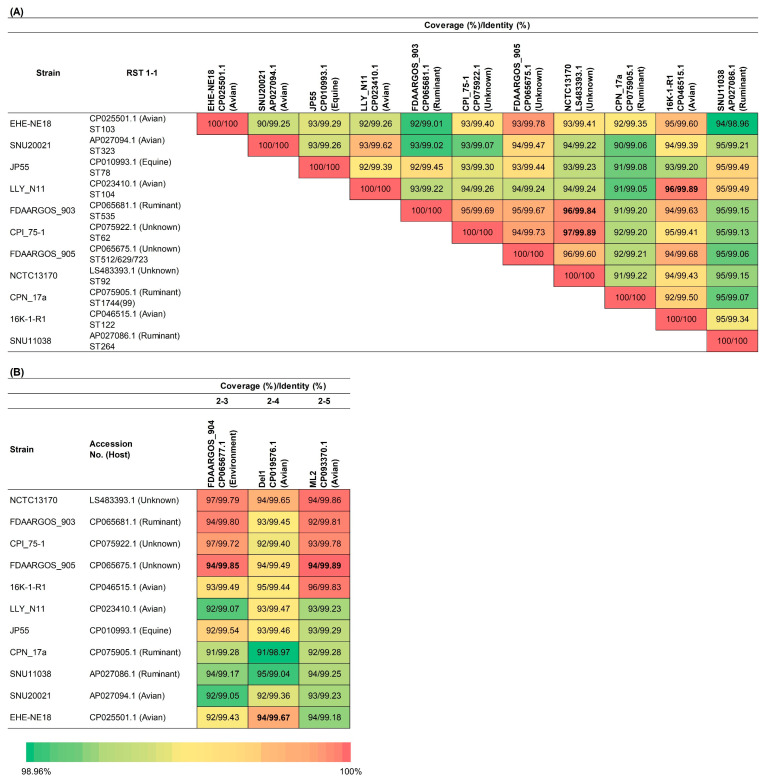
Heatmap representing pairwise sequence identity (%) between RST 1-1 and RSTs 2-3, 2-4, and 2-5. The identity values were calculated based on full-length sequences obtained from the NCBI GenBank database. The color gradient indicates the degree of sequence similarity. (**A**): Heatmap showing identity values among RST 1-1 sequences. (**B**): Heatmap showing sequence identity values between RST 1-1 and RSTs 2-3, 2-4, and 2-5. RST 2-3 and RST 2-5 showed high similarity to the same RST 1-1 isolates, whereas RST 2-4 was most similar to a different RST 1-1 isolate. This visualization highlights potential evolutionary relationships between RSTs. Bold numbers indicate identity values highlighted in the Section 3 as key findings.

To trace potential evolutionary pathways from RST 0 to directly connected RSTs, we compared the genome coverage and identity between strains. In our previous study, we found that values of ≥94% coverage and ≥99.8% identity indicated a close evolutionary relationship. Due to the limited availability of complete genomes across all RSTs, we focused on RST 1-1, which had a greater number of sequences. Several RST 1-1 strains showed high similarity to one another (Figure 3A); for instance, NCTC13170 exhibited 96% coverage and 99.84% identity with FDAARGOS_903 while showing 97% coverage and 99.89% identity with CPI_75-1. LLY_N11 also showed 96% coverage and 99.89% identity with 16K-1-R1.

Among the RST 2 and 3 types branching from RST 1-1 in the network, complete genome sequences were available only for strains in RST 2-3, RST 2-4, and RST 2-5. These sequences were subsequently compared with those of RST 1-1 strains (Figure 3B). The RST 2-3 strain showed the highest coverage and identity with FDAARGOS_905 (94/99.85%). The RST 2-4 strain was most similar to EHE-NE18 (94%/99.67%), while the RST 2-5 strain showed the highest coverage and identity with FDAARGOS_905 (94%/99.89%).

Given that genomic diversity can exist among isolates within the same RST, a shared RST alone is not sufficient to indicate a direct progenitor–progeny relationship. However, such relationships may exist between strains in RSTs that are directly connected in the network, indicating that network connectivity is a necessary condition for evolutionary inference. While direct progenitor–progeny relationships have not yet been conclusively established, the data suggest that these RSTs may be closely related.

### 3.6. Evolutionary Relationships Between Different Genera and Species of Gram-Positive Bacteria with rpoB Genes of Different Lengths

The length of the *rpoB* gene tends to increase from Gram-positive to Gram-negative bacteria. Accordingly, we analyzed and compared the *rpoB* gene lengths across various genera and species of Gram-positive bacteria (Supplementary Table S9). Among the bacteria analyzed, *C. perfringens* exhibited a relatively longer *rpoB* gene than most others, with *C. chauvoei* and *C. tetani* sharing the same length (3711 bp). Notably, *C. difficile* and *Bacillus anthracis* had even longer *rpoB* sequences, measuring 3717 bp and 3742 bp, respectively.

*Clostridium* species and other genera with the same *rpoB* length as *C. perfringens* are listed in Supplementary Table S10. Among these, *C. baratii* displayed the highest *rpoB* sequence identity (43.34–67.75%) with *C. perfringens*.

However, due to the limited availability of high-quality genome sequences for these species, a direct correlation between *rpoB* sequence identity and overall genomic coverage or identity could not be established.

### 3.7. Prophage Typing and CRISPR-Cas System Profile in C. perfringens

Prophage typing revealed that most isolates lacked detectable prophages, consistent with the stability of ancestral RSTs. Only the two RST 2-17 isolates exhibited mPPT 15, while the in silico analysis of the complete GenBank sequences showed variability in mPPT across different RSTs (Supplementary Table S6). Among the three RST 0 sequences, one strain carried mPPT 17 (strain CPI 18-1b, CP075981.1), which was also detected in four out of nine RST 1-1 sequences. Within RST 2, mPPT 17 was present in RSTs 2-4, 2-7, 2-10, and 2-17, and it was also identified in RSTs 4-3, 8-2, 8-3, and 11-1. Similarly, mPPT 12 and 23, which were found in another RST 0 strain (strain 721/84, CP075953.1), were also detected in RSTs 1-1, 1-4, 2-19, and 11-6. Although the limited sample size prevents definitive conclusions, the data suggest that prophage acquisition may be linked to the evolutionary divergence of *C. perfringens* from consensus RSTs.

Among the five complete genomes analyzed in this study, SNUVG (RST 0) and SNU20021 (RST 1-1) harbored one and two CRISPR-Cas systems, respectively (Table 2). SNUVG harbors a type I-B CRISPR-Cas system of the Tneap subtype (CRT 1), first characterized in *Thermotoga neapolitana*, comprising 28 spacers. In contrast, SNU20021 harbors two CRISPR arrays (CRT 2 and CRT 3), both of which were classified as Type I-B/Hmari based on their repeat consensus sequences, a family originally described in *Haloarcula marismortui.* [42]. Despite their different origins, these two subtypes are often collectively referred to as the Tneap–Hmari system due to their conserved *cas* gene architectures.

The spacer sequences identified in these CRISPR-Cas systems are listed in Supplementary Table S7. As is typical of CRISPR array dynamics, new spacers are generally integrated at the 5′ end, while older, non-functional spacers may be randomly deleted over time [43]. Notably, spacer 28 from the CRT 1 system was consistently found in strains representing RST 0, RST 1, RST 2, and even RST 16 (Supplementary Table S6), suggesting that this spacer may play a conserved and functionally significant role.

The spacer origin analysis also revealed that, in SNUVG (CRT 1), 14 of the 19 spacers targeted phage DNA, 4 targeted plasmids, and 1 targeted a polymerase polyprotein. Similarly, in SNU20021, 30 of 36 CRT 2 spacers targeted phages, and 6 targeted plasmids. These findings indicate that bacteriophages are the primary targets of CRISPR-Cas systems in *C. perfringens*, reflecting a defense strategy against horizontal gene transfer mediated by mobile genetic elements [44].

### 3.8. Comparison of Genetic Similarity Between Plasmids

The nucleotide sequences of the plasmids identified in the five *C. perfringens* strains examined in this study are summarized in Supplementary Table S8. These plasmids originated from distinct RSTs: pSNUVG (RST 0), pSNU20021b (RST 1-1), pSNU11038a (RST 1-1), pSNU21005b (RST 2-17), and pSNU61035a (RST 2-17). Despite their diverse origins, the plasmids exhibited relatively high sequence identity (76.14-99.99%). Among them, pSNUVG, pSNU11038a, and pSNU20021b shared two key genes—T4CP and *cpb2* (Table 3). T4CP encodes the type IV coupling protein, a component of the type IV secretion system (T4SS), which mediates plasmid conjugation, protein translocation, and virulence factor delivery [45].

Notably, pSNU11038a also contained the complete T4SS operon, whereas pSNU20021b carried the origin of transfer (oriT), which serves as a recognition and cleavage site for relaxase, initiating plasmid mobilization and promoting horizontal gene transfer (HGT) [46]. The conjugation potential of these plasmids was inferred based on the presence of core conjugation-related genes (e.g., T4CP, T4SS, and oriT), which together suggest a genetic capacity for horizontal transfer, although experimental validation was beyond the scope of this study.

Insertion sequence (IS) elements were also found near the *cpb2* gene in these plasmids, but their families differed. pSNU20021b and pSNU11038a harbored IS4/IS231 elements, whereas pSNUVG contained an unrelated IS256 element. Additionally, pSNUVG uniquely carried an anti-CRISPR (AcrIIA7), known to inhibit CRISPR-Cas systems and facilitate the retention of foreign DNA [47]. The presence of AcrIIA7 in the RST 0 strain may reflect an evolutionary strategy to tolerate or incorporate exogenous genetic material, potentially enhancing its adaptability.

## 4. Discussion

Following the 2011 ban on antimicrobial growth promoters (AGPs) in Republic of Korea [48], poultry necrotic enteritis (NE) cases increased, along with therapeutic antibiotic use. This shift in antibiotic usage patterns has been linked to both higher resistance rates and increased virulence. Specifically, the prevalence of the *cpb2, netB*, and *tpeL* toxin genes has been reported to have risen after AGP discontinuation. In our study, we identified *cpb2*-positive isolates; however, no *tpeL*-positive isolates were detected. This finding is consistent with prior studies indicating that *tpeL* is exclusively present in NE-affected birds and absent in healthy individuals. The absence of *tpeL*-positive isolates in our study suggests that the primary cause of mortality in our sampled birds was not necessarily *C. perfringens*.

The *cpb2* gene encodes the β2 toxin, which plays a critical role in *C. perfringens* pathogenicity by disrupting cell membrane integrity and tight junction proteins [49]. Despite its importance, *cpb2* is often omitted from standard PCR-based typing schemes [2]. Previous studies have reported *cpb2* prevalence rates in healthy poultry ranging from 57.7% to 90% [48,50]; in our study, 89.47% of isolates were *cpb2*-positive. This high prevalence rate implies that the environmental conditions and host immune status may significantly influence toxin gene expression. Although aminoglycoside treatment has been linked to the induction of *cpb2* expression in horses [51], our lack of treatment history data limited conclusions regarding whether expression was induced in our isolates. A previous analysis of *C. perfringens* strains from human food poisoning cases showed that all isolates were type A and carried the *cpb2* toxin, similarly to those identified in our study [52]. Therefore, the potential of our isolates to cause food poisoning in humans needs to be demonstrated through comparisons of the RSTs, STs, and genomes with those in human isolates.

In addition to bacterial virulence factors, co-infections with immunosuppressive viral pathogens such as IBDV, CAV, FAdV-4, and ALV disrupt host immune responses, facilitating bacterial colonization and exacerbating disease severity [53,54,55,56]. Chicken interferon-α (ChIFN-α) and ChIFN-γ transcription is disrupted by both IBDV and CAV, allowing pathogens to evade immune surveillance [54]. FAdV-4, known to cause hydropericardium syndrome in birds, results in lymphocyte apoptosis in lymphoid organs and growth impairments in the thymus and bursa. FAdV-4 also causes a reduced humoral immune response, with the upregulation of both caspase-3 mRNA and the *bax* and *p53* genes, both of which are pro-apoptotic genes that induce lymphocyte apoptosis [53]. In ALV, the p27 capsid protein inhibits TNF-α expression in macrophages and causes immunosuppression [56]. These observations highlight the importance of integrated pathogen control approaches in effectively managing co-infections and reducing the *C. perfringens* load and the possibility of food poisoning.

The slow evolutionary rate of *C. perfringens* is likely attributable to its spore-forming properties. Throughout its life cycle, the bacterium frequently persists in a dormant state as an environmental contaminant until exposure to gastric acid within a host triggers a transition to its active form. In contrast, the original hosts of *E. coli* and *S. aureus* are humans [9,13]. Because *E. coli* and *S. aureus* actively replicate across diverse environments, whereas *C. perfringens* spends much of its existence in dormancy, the latter experiences reduced selective pressure for rapid evolution. The unexpectedly high frequencies of RST 0 and RST 1-1 in our study further reflect this slow evolutionary rate. The anaerobic conditions required for *C. perfringens* growth are provided by the rumens of ruminants and the well-developed ceca of chickens, horses, and rabbits, making these animals natural reservoirs. Nevertheless, it should be noted that avian isolates dominate publicly available genome databases, and this overrepresentation may introduce sampling biases in host- and region-specific RST frequencies. Therefore, the inference that chickens serve as a major reservoir should be interpreted with caution. Differences in host, geography, and collection year across datasets could influence RST distributions, and future studies incorporating balanced sampling or weighted analyses will be required to validate these findings.

To better understand the genomic basis underlying pathogen evolution and transmission, we employed *rpoB* sequence typing (RSTing). A previous study classified *E. coli* strains using RST typing and conducted further subgrouping to investigate bacterial evolution within specific RST groups [13]. By analyzing the genomic coverage and sequence identity, the mentioned study identified significant genetic variation even within the same RST and inferred possible evolutionary trajectories, showing how a specific group within an RST led to the emergence of higher-numbered RSTs. Although applying the same approach to *C. perfringens* was challenging due to the limited number of isolates, our results revealed that, even within the same RST, certain strains exhibited high identity. By comparing the identities between these strains and their diverging counterparts, it was possible to determine both commonalities and differences. Despite the limitations of our dataset, we confirmed that certain RST 1-1 subgroups exhibited distinct genomic coverage and identity despite belonging to the same RST, thus providing deeper insights into the evolutionary trajectory of *C. perfringens*.

Pan-genome analyses have provided new insights into the evolution of species and the pathogenicity of bacteria [11]. Nevertheless, simpler and more cost-effective first-line approaches remain necessary. Previous studies have reported that the diversity indices of MLST and RSTing are comparable in *Salmonella* serotypes [32], *S. aureus*, and *E. coli*. In the case of *C. perfringens*, however, MLST demonstrated a stronger discriminatory capacity. These findings suggest that applying MLST after RSTing may be informative for the molecular epidemiological investigation of *C. perfringens* isolates, although the optimization of primer sets is required because amplification failures were observed for some housekeeping genes [26].

Moreover, our detection of the *cpb2* gene in both poultry and cattle isolates suggests the plausibility of interspecies transmission. The comparative plasmid analysis, demonstrating shared insertion sites and high sequence identities between chicken and cattle RST 2-17 isolates, further supports this hypothesis. This finding is particularly relevant considering the potential transmission routes, such as the use of poultry-derived products in cattle feed and the contamination of underground water with mixed manure-based fertilizers (from chickens, pigs, and cattle), which may be used as drinking water for domestic animals.

Network-based RST analysis has also revealed evolutionary links among closely related species. In our previous study, we identified close relationships between *E. coli* and *Escherichia fergusonii*, *E. albertii*, and *E. marmotae*, which share the same *rpoB* gene length [13]. These species were classified within the *E. coli* RSTing scheme and were connected to the evolutionary trajectory of *E. coli*, underscoring the value of RSTing in exploring speciation events. Extending the scope to *Clostridium*, we observed that *C. baratii* and *C. botulinum*, despite being taxonomically distinct, shared identical *rpoB* gene lengths with *C. perfringens* (Supplementary Table S10). While *C. baratii* is not taxonomically related to *C. botulinum* [57], it harbors the botulinum neurotoxin (BoNT) gene cluster, specifically producing type F botulinum toxin, which is implicated in rare cases of infant botulism [58,59]. Both species also occupy overlapping ecological niches in soil [60], and their similar *rpoB* lengths to *C. perfringens* suggest shared ancestry.

Further analysis of the *rpoB* sequence length, whole genome size, and GC content across various Gram-positive bacteria revealed that an increase in the *rpoB* sequence length does not correlate with the whole-genome length (Supplementary Table S9). Since it is known that low-GC clades are interspersed among high-GC clades within the same bacterial species [61], this characteristic does not reliably indicate an evolutionary direction. Nevertheless, considering that the type strains *Clostridium chauvoei* [62] and *Clostridium tetani* [63] share similar *rpoB* lengths, we can infer that these two species are genetically closely related. Similarly, within the *Staphylococcus* genus, *S. aureus* and *S. epidermidis* possess identical *rpoB* lengths, suggesting a shared ancestral lineage. Collectively, these findings support the *rpoB* length as an additional metric for the assessment of evolutionary relationships within bacterial taxa.

Regarding bacterial defense mechanisms, the presence of phage-derived CRISPR spacers in RST 0 isolates indicates prolonged ancestral exposure to phage attacks. This accumulation of phage-derived spacers likely reflects an adaptive mechanism to combat future infections, reinforcing the idea that CRISPR systems play a crucial role in bacterial evolutionary dynamics and host–pathogen interactions [64]. Furthermore, our network analysis also identified a connection between RST 1-1 (e.g., LLY_N11, CP023410.1; CPI 75-1, CP075922.1) and RST 2-3 (FDAARGOS_904, CP065677.1). The presence of CRT 1 spacer 28 in both RSTs supports this observed relationship, suggesting a potential evolutionary link between the mentioned RSTs.

Notably, CRT spacer 28 exhibited 86.11% sequence identity to a phage protein (Supplementary Table S7). A previous study in *E. coli* reported spacer targets predominantly originating from phages (57%), followed by plasmids (31%) and chromosomal regions (12%) [65]. Our findings are consistent with previous reports, as 57.7% (45/78) of CRT 1 and CRT 2 spacers in our dataset were phage-derived, whereas only 15.4% (12/78) were plasmid-derived and none originated from chromosomal sequences. These results suggest that the CRISPR-Cas systems in these strains predominantly target phage elements, potentially shaping genomic evolution.

## 5. Conclusions

In conclusion, we applied RSTing together with CRISPR profiling, mPPT, and MLST to investigate the genetic diversity and evolutionary features of *C. perfringens*. Although the limited sample size prevented definitive conclusions, our analyses indicate that chickens may serve as an important reservoir, with evidence of potential interspecies circulation between chickens and cattle through shared RST 2-17 isolates. Rather than serving as a standalone framework, RSTing, when combined with complementary approaches such as mPPT, CRISPR, and MLST, can provide a practical supplementary tool for molecular epidemiology and evolutionary studies of *C. perfringens*.

## Figures and Tables

**Table 1:** Characteristics of *C. perfringens* isolates used in this study.

Host	Strain	Toxinotype	RST	ST	Year	Region ^a^	Source ^b^	Other Pathogens ^c^
Chicken	SNU20021	A	1-1	323	2020	JJ	Layer/CF	CAV, ILTV, PV, FadV, IBV
	SNU20055	A	1-1	21	2020	CN	Broiler/CF	FadV, IBDV, Coccidiosis, *E. coli*
	SNU21002	A	1-1	UT1	2021	KB	Broiler/CF	FadV, IBV, IBDV, *E. coli*
	SNU21004	A	4-1	179	2021	CN	Layer/CF	CAV, FadV, IBV, IBDV
	SNU21005	A	2-17	277	2021	CN	Broiler/CF	FadV, IBV
	SNU21006	A	1-1	iUT2	2021	JJ	Layer/CF	FadV, IBV
	SNU21008	A	2-16	587	2021	KN	Layer/CF	CAV, IBV, *E. coli*
	SNU21011	A	1-1	UT3	2021	KB	Broiler/CF	IBV, ALV, ATV
	SNU21012	A	2-17	UT4	2021	CN	Breeder/CF	FadV, IBDV, *E. coli*
Cattle	SNU124	A	7-2	601	nk	KW	F	nd
	SNU513	E	5-9	iUT4	nk	KW	F	nd
	SNU1045	A	0	iUT1	nk	KW	F	nd
	SNU7253-1	E	5-9	iUT4	nk	KW	F	nd
	SNU11038	A	1-1	294	nk	KW	F	nd
	SNU11040	A	1-4	iUT3	nk	KW	F	nd
	SNU17037	A	1-1	UT2	nk	KW	F	nd
	SNU61035	A	2-17	277	nk	KW	F	nd
	SNUBA1100001	A	1-1	nd	nk	KW	F	nd
	SNUVG	A	0	885	nk	KW	VD	nd

^a^ JJ, Jeju; CN, Chungcheongnam-do; KB, Gyeongsangbuk-do; KN, Gyeongsangnam-do; KW, Kangwon-do. ^b^ CF, cecal feces; F, feces; VD, vaginal discharge. ^c^ CAV, chicken anemia virus; ILTV, infectious laryngotracheitis virus; PV, pox virus; FadV, fowl adenovirus; IBV, infectious bronchitis virus; IBDV, infectious bursal disease virus; ALV, avian leukosis virus; ATV, avian tremovirus; nk, not known; nd, not determined.

**Table 2:** Comparative genomics findings for selected *C. perfringens* strains.

	SNUVG	SNU20021	SNU11038	SNU21005	SNU61035
ST	885	323	294	277	277
Accession number	AP027100.1	AP027094.1	AP027086.1	AP027090.1	AP027082.1
Origin	Holstein vaginal discharge	Chicken	Holstein feces	Chicken	Holstein feces
RST	0	1-1	1-1	2-17	2-17
mPPT	-	-	-	15	15
Toxinotype	A	A	A	A	A
Total length (bp)	3,300,601	3,455,495	3,222,972	3,635,260	3,623,803
GC content (%)	28.5	28.3	28.4	28.4	28.4
No. of CDSs	2913	3120	2835	3362	3349
No. of rRNAs	30	30	30	29	31
No. of tRNAs	94	94	94	95	97
CRISPRS (spacer)	I-B/Tneap (28)	I-B/Hmari (50) I-B/Hmari (39)	-	-	-
Coding ratio (%)	83.4	83.5	83.1	83.3	83.4
Plasmid 1	pSNUVG *	pSNU20021a	pSNU11038a *	pSNU21005a	pSNU61035a
	48,130 bp	94,966 bp	77,679 bp	69,347 bp	63,436 bp
	(AP027101.1)	(AP027095.1)	(AP027087.1)	(AP027091.1)	(AP027083.1)
plasmid 2		pSNU20021b *	pSNU11038b	pSNU21005b	pSNU61035b
		69,834 bp	52,631 bp	63,988 bp	47,638 bp
		(AP027096.1)	(AP027088.1)	(AP027092.1)	(AP027084.1)
Plasmid 3		pSNU20021c	pSNU11038c	pSNU21005c	pSNU61035c
		14,273 bp	14,755 bp	47,639 bp	41,064
		(AP027097.1)	(AP027089.1)	(AP027093.1)	(AP027085.1)
Plasmid 4		pSNU20021d			
		10,522 bp			
		(AP027098.1)			
Plasmid 5		pSNU20021e			
		3842 bp			
		(AP027099.1)			
No. of genomic islands (GI) ^a^	3	5	2	9	9

^a^ Prediction was performed using IslandViewer 4 (including IslandPick, IslandPath-DIMOB, SIGI-HMM, and Islander). * indicates plasmids that harbor the *cpb2* gene.

**Table 3:** Comparison of gene content of *cpb2*-harboring plasmids.

Plasmid (RST/Host)	Conjugative Transfer			Virulence	Transposable Element	Anti-CRISPR
	oriT	T4CP	T4SS	*cpb2*	Insertion Site Family	
pSNUVG (RST 0/Cattle)	–	+	–	+	IS256	AcrllA7
pSNU11038a (RST 1-1/Cattle)	–	+	+	+	IS4/IS231	
pSNU20021b (RST 1-1/Chicken)	+	+	–	+	IS4/IS231	

‘+’ = present; ‘–’ = absent.

## Data Availability

The whole-genome sequences generated in this study have been deposited in the NCBI GenBank database under the accession numbers AP027082.1, AP027086.1, AP027090.1, AP027094.1, and AP027100.1. The *rpoB* gene sequences are available under accession numbers LC885342–LC885355. All sequences are publicly accessible at https://www.ncbi.nlm.nih.gov/.

## Supplementary Materials

The following supporting information can be downloaded at: https://www.mdpi.com/article/10.3390/microorganisms13122768/s1, Table S1: Primer sets used in this study; Table S2: Genetic profiles of toxin genes in *Clostridium perfringens* strains isolated in this study; Table S3: Isolate information from the NCBI GenBank^®^ and pubMLST databases; Table S4: *rpoB* sequence types (RSTs) of *Clostridium perfringens* strains; Table S5: Assignment of sequence types (STs) and amplification results for eight housekeeping genes in *Clostridium perfringens* strains; Table S6: Sequences positive for RSTs and mPPT from the GenBank database; Table S7: Targets of CRISPR spacers from strains isolated from this study; Table S8: Pairwise sequence coverage and identity (%) of plasmids identified in this study; Table S9: Comparison of *rpoB* gene size, whole genome size, and GC content in various Gram-positive type strains; Table S10: Gram-positive bacteria with identical *rpoB* lengths to *C. perfringens*. Figure S1: Maximum-likelihood phylogeny of *Clostridium perfringens* strains based on full-length *rpoB* sequences.

## Abbreviations

The following abbreviations are used in this manuscript:

AGP	Antimicrobial Growth Promoter
ALV	Avian Leukosis Virus
BoNT	Botulinum Neurotoxin
*C. perfringens*	*Clostridium perfringens*
CAV	Chicken Anemia Virus
CRISPR	Clustered Regularly Interspaced Short Palindromic Repeats
FAdV-4	Fowl Adenovirus Serotype 4
HGT	Horizontal Gene Transfer
IBDV	Infectious Bursal Disease Virus
NE	Necrotic Enteritis
PAM	Protospacer Adjacent motif
PCR	Polymerase Chain Reaction
RST	*RpoB* Sequence Type
