# Supplementary figures and images for: An rpoB Sequence Type Network as a Framework for the Evolutionary Investigation of Clostridium perfringens
Source: Microorganisms. 2025 Dec 4;13(12):2768. doi: 10.3390/microorganisms13122768 (PMC12736292; doi:10.3390/microorganisms13122768)

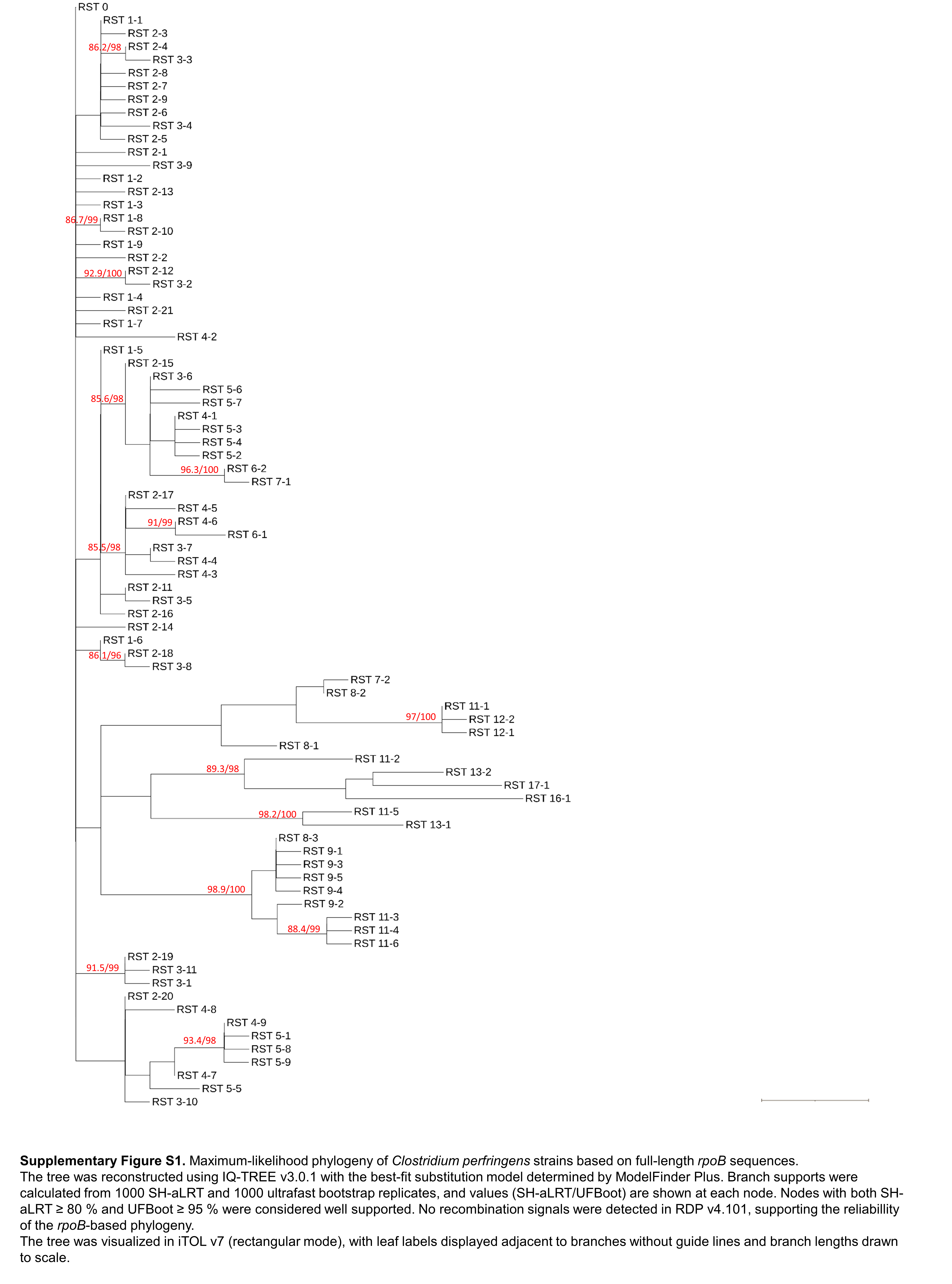

Supplement: Supplementary file 1 [file microorganisms-13-02768-s001.zip › Supplementary Figure S1.tif]
